# Fluoxetine exposure during adolescence increases preference for cocaine in adulthood

**DOI:** 10.1038/srep15009

**Published:** 2015-10-09

**Authors:** Sergio D. Iñiguez, Lace M. Riggs, Steven J. Nieto, Katherine N. Wright, Norma N. Zamora, Bryan Cruz, Arturo R. Zavala, Alfred J. Robison, Michelle S. Mazei-Robison

**Affiliations:** 1 Department of Psychology, The University of Texas at El Paso, El Paso, TX, USA; 2 Department of Psychology, California State University, San Bernardino, CA, USA; 3 Department of Biomedical Sciences, Florida State University, FL, USA; 4 Department of Psychology, California State University, Long Beach, CA, USA; 5 Department of Physiology, Michigan State University, Michigan, MI, USA

## Abstract

Currently, there is a high prevalence of antidepressant prescription rates within juvenile populations, yet little is known about the potential long-lasting consequences of such treatments, particularly on subsequent responses to drugs of abuse. To address this issue at the preclinical level, we examined whether adolescent exposure to fluoxetine (FLX), a selective serotonin reuptake inhibitor, results in changes to the sensitivity of the rewarding properties of cocaine in adulthood. Separate groups of male c57bl/6 mice were exposed to FLX (0 or 20 mg/kg) for 15 consecutive days either during adolescence (postnatal days [PD] 35–49) or adulthood (PD 65–79). Twenty-one days after FLX treatment, behavioral responsivity to cocaine (0, 2.5, 5, 10, or 20 mg/kg) conditioned place preference was assessed. Our data shows that mice pretreated with FLX during adolescence, but not during adulthood, display an enhanced dose-dependent preference to the environment paired with cocaine (5 or 10 mg/kg) when compared to age-matched saline pretreated controls. Taken together, our findings suggest that adolescent exposure to FLX increases sensitivity to the rewarding properties of cocaine, later in life.

Pediatric depression has only recently become well recognized. Today, mood disorders are diagnosed in up to 9% of children and adolescents, and if left untreated, may result in negative consequences that extend into adulthood^1^. For instance, it is estimated that children and adolescents who suffer from major depressive disorder (MDD) are more likely to develop conduct-, anxiety-, and substance use related illnesses^2^. Consequently, this has resulted in a dramatic increase in the prescription of antidepressants to populations under 20 years of age^3^. Despite heightened rates of antidepressant use, little is known about the long-term neurobiological adaptations that may result from antidepressant treatment during periods prior to adulthood^4^.

Preclinical studies indicate that early-life exposure to fluoxetine (FLX), a selective serotonin reuptake inhibitor (SSRI), results in long-lasting neurobehavioral alterations in adulthood^4^,^5^. Specifically, FLX exposure during juvenile stages of development induces a long-lasting and complex behavioral response, wherein rodents exhibit decreases in sensitivity to inescapable stressors^6^,^7^,^8^, along with increases in sensitivity to anxiety-inducing situations^5^,^9^. This is not surprising given that neuronal adaptations occur during the adolescent period of development^10^, which have been correlated, at least in part, with responsiveness to emotional- and reward-related stimuli under normal conditions^11^. Interestingly, exposure to FLX during adolescence has also been reported to induce an enduring heightened sensitivity to natural rewards (i.e., sucrose^5^) – suggesting that such pharmacological treatment may also influence the development of brain pathways associated with responsiveness to drug-associated reward. This is plausible given that early-life exposure to a variety of psychotropic drugs, including antidepressants and/or stimulants, have been found to enhance sensitivity to drugs of abuse in adulthood^12^,^13^,^14^. Thus, the goal of this investigation is to examine whether FLX exposure, during adolescence specifically, results in long-lasting increases in sensitivity to the rewarding effects of cocaine. To this end, we selected the conditioned place preference (CPP) paradigm, as it has been widely utilized to assess the rewarding and/or aversive properties of abused drugs^15^.

## Results

### Long-term effects of FLX exposure during adolescence on cocaine CPP

**Figure 1:**
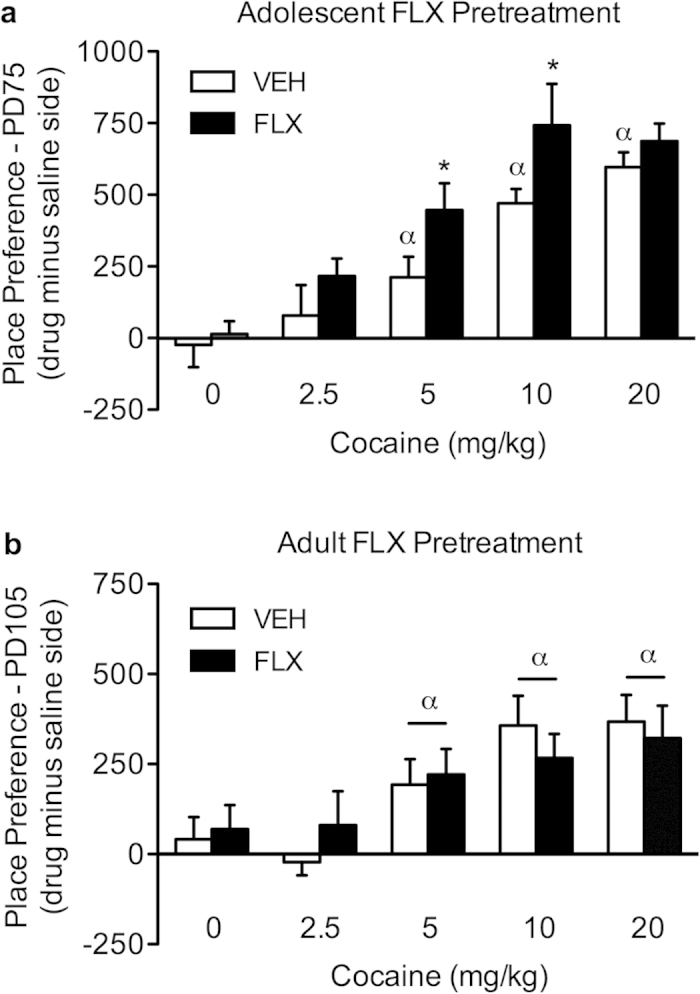
Enduring effects of fluoxetine (FLX; 20 mg/kg) exposure on cocaine-induced place conditioning. (**a**) Three-weeks after adolescent antidepressant exposure (postnatal day [PD]-75), FLX-pretreated mice displayed enhanced sensitivity to 5 and 10 mg/kg cocaine, when compared to vehicle (VEH)-pretreated mice (n = 8–11 per group; *p < 0.05). (**b**) Conversely, adult FLX-pretreatment did not influence preference for the cocaine-paired side, three-weeks after antidepressant exposure (PD 105; n = 7–10 per group; *p* > 0.05). Regardless of age, VEH-and FLX-pretreated mice displayed reliable conditioning to cocaine (^α^*p* < 0.05). *Within cocaine group comparison (*p* < 0.05). ^α^Significantly different when compared to age-matched controls conditioned to saline (*p* < 0.05).

Figure 1a shows the enduring effects of adolescent FLX exposure on cocaine (0, 2.5, 5, 10, or 20 mg/kg) CPP in adulthood (N = 90). Time spent in the cocaine-paired compartment varied as a function of adolescent FLX-pretreatment (main effect: *F*_(1,80)_ = 9.15, *p* < 0.003), and cocaine exposure in adulthood (post-treatment main effect: *F*_(4,80)_ = 22.30, *p* < 0.0001). Importantly, neither VEH- (n = 10) nor FLX-pretreatment (n = 9) resulted in preference for any of the compartments when animals were conditioned to saline (*p* > 0.05). Similarly, no preference for either compartment was observed in mice conditioned to the lowest dose of cocaine (2.5 mg/kg), regardless of VEH (n = 8) or FLX (n = 8) exposure during adolescence (*p* > 0.05). Conversely, VEH-pretreated mice conditioned with 5 (n = 11), 10 (n = 8), and 20 (n = 8) mg/kg cocaine displayed reliable conditioning, when compared to VEH-pretreated/saline-conditioned mice (*p* < 0.05, respectively). Planned comparisons indicated that FLX pretreatment resulted in reliable conditioning to the compartment paired with 5 (n = 11), 10 (n = 9), and 20 (n = 8) mg/kg cocaine, when compared to FLX-pretreated/saline-conditioned mice. Interestingly, FLX-pretreated mice conditioned to 5 and 10 mg/kg cocaine spent significantly more time in the drug-paired compartment when compared to VEH-pretreated mice receiving the same doses of cocaine in adulthood (*p* < 0.05, respectively) – suggesting that adolescent FLX-pretreatment increased the incentive value of cocaine in adulthood. Notably, no differences in general locomotor activity (distance traveled in cm), as a function of adolescent FLX pretreatment (postnatal day [PD] 35–49), were observed during the preconditioning phase (PD 70, *p* > 0.05, data not shown).

### Long-term effects of FLX exposure in adulthood on cocaine CPP

To examine whether the enduring increase in sensitivity to the rewarding properties of cocaine is dependent on age of FLX exposure (adolescence vs. adulthood), we treated adult mice with FLX for 15 consecutive days (PD 65–79), and examined their behavioral responses to cocaine CPP three-weeks later (PD 105; N = 93). As shown in Fig. 1b, adult pretreatment with FLX did not result in a long-lasting increase in sensitivity to cocaine, as was observed with adult mice pretreated with FLX during adolescence (Fig. 1a). Results indicate that the time spent in the cocaine-paired compartment varies as a function of cocaine dose (post-treatment main effect: *F*_(4,83)_ = 6.46, *p* < 0.0001), but not as a function of FLX-pretreatment (main effect: *p* > 0.05), or their interaction (FLX-pretreatment × cocaine post-treatment: *p* > 0.05). Mice conditioned to saline (n = 10 per group) or 2.5 mg/kg cocaine (n = 7 − 10 per group) did not display a preference for either compartment (*p* > 0.05, respectively). On the other hand, mice conditioned to 5 (n = 7 − 10 per group), 10 (n = 10 per group), and 20 (n = 9 − 10 per group) mg/kg cocaine spent significantly more time in the cocaine-paired side, regardless of antidepressant pre-exposure (VEH vs. FLX), when compared to saline-conditioned mice (*p* < 0.05, respectively). Lastly, no differences in distance traveled (cm), as a function of antidepressant exposure in adulthood (PD 65–79), were observed between the groups during the preconditioning phase (PD 100, *p* > 0.05, data not shown).

## Discussion

This study was designed to examine whether FLX, a SSRI that is increasingly prescribed to adolescents, results in altered sensitivity to the rewarding properties of cocaine later in life. This approach was taken because previous reports show that juvenile FLX exposure results in long-lasting increases in sensitivity to natural rewards, namely a sucrose solution^5^,^7^. Here, we report that exposure to FLX during adolescence, but not adulthood, enhances responsiveness to the rewarding properties of cocaine later in life, as measured in the CPP paradigm.

Mice pretreated with FLX during adolescence (PD 35–49) showed increased sensitivity to environments paired with moderately low doses of cocaine (5 and 10 mg/kg), when compared to mice that receive the same doses of cocaine, yet pretreated with saline during adolescence (Fig. 1a). Conversely, animals pretreated with FLX in adulthood (PD 65–79) did not show an enduring enhanced sensitivity to cocaine 21 days after treatment, when compared to their respective saline-treated age-matched controls (Fig. 1b). Importantly, no differences in general locomotor activity were observed as a function of antidepressant pretreatment, regardless of whether FLX was administered during adolescence or adulthood, thus suggesting that the responses to cocaine were not attributed to FLX-induced alterations in exploratory behavior^5^,^7^. When considered together, these data suggest that immature neuronal systems associated with reward and motivation are susceptible to alterations induced by FLX treatment^6^,^16^,^17^. This FLX-induced increase in sensitivity to drug reward resembles that of preadolescent rats exposed to FLX (PD 20–34), and tested on cocaine place conditioning two months after treatment^7^. Here, we extend these findings to mid-adolescence (PD 35–49), the developmental stage where the first episode of clinical depression is most often reported^18^.

The neurobiological mechanisms underlying the FLX-induced increase in sensitivity to cocaine are unknown. Because CPP is contingent upon an animal associating the rewarding/aversive properties of a drug with environmental cues, areas of the brain that are essential for memory associations of contextual stimuli, such as the ventral tegmental area and hippocampal formation^19^,^20^,^21^,^22^, are likely to play a role in the behaviors observed. In particular, the hippocampus has been shown to be a key mediator for the acquisition and expression of cocaine CPP^20^,^23^. In addition to being crucial for drug-related contextual memories^24^,^25^,^26^, the hippocampus has also been a site of FLX-induced alterations of signaling molecules associated with antidepressant efficacy^27^. Within this brain region, FLX has been shown to increase levels of brain derived neurotrophic factor^28^, as well as several of its downstream signaling targets. Particularly, the extracellular signal-regulated kinase (ERK) is essential to various forms of learning and memory^24^,^29^, in addition to mediating behavioral responses to cocaine^19^,^30^, and mood-related behaviors^31^,^32^,^33^. Therefore, it is conceivable that FLX-induced behavioral responses to reward may be mediated by long lasting adaptations of ERK signaling within the hippocampus. Alternatively, adolescent FLX exposure has recently been found to induce a long-lasting upregulation of the serotonin transporter (SERT)^16^, which in turn, may underlie the observed facilitated cocaine CPP, given that cocaine has a high affinity for SERT^34^,^35^,^36^. Accordingly, future studies will be needed to thoroughly assess these hypotheses.

Overall, the present study demonstrates that chronic adolescent exposure to FLX increases sensitivity to cocaine in adulthood. It is imperative to note, that the FLX-induced effects observed in this study were from animals that were not purposefully stressed and that FLX exposure in models of juvenile depression may yield different results^37^,^38^. For example, it would be interesting to examine how co-exposure to FLX and social defeat stress, during the adolescent stage of development, influences the rewarding properties of cocaine in adulthood. Another limitation is that we did not include female subjects in our experimental design, restricting the interpretability of the present data to the clinical setting, where twice as many females, when compared to males, are diagnosed with mood-related disorders, and thus are more likely to be prescribed with antidepressant medications.

## Methods

### Subjects

A total of 183 male mice, of the c57bl/6 strain, were used in this investigation. Mice were obtained from the Department of Psychology Mouse-Breeding Colony at California State University San Bernardino (CSUSB). Mice were housed (3–4 per cage) in standard polypropylene cages containing wood shavings and placed on a 12-h light/dark cycle (lights on at 7:00 A.M.) with unrestricted access to food and water. All experiments were conducted in compliance with the National Institutes of Health *Guide for the Care and Use of Laboratory Animals*, and with approval of the Institutional Animal Care and Use Committee at CSUSB.

### Drugs and experimental design

**Figure 2:**
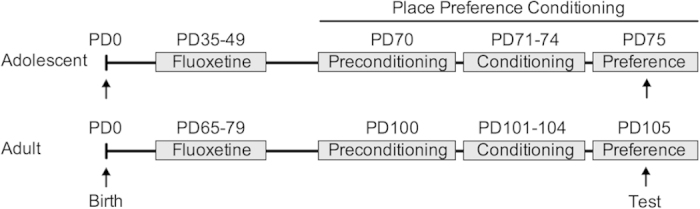
Timeline of developmental fluoxetine (FLX) treatment and cocaine place conditioning procedures. Separate groups of adolescent (postnatal day [PD]-35) and adult (PD 65) male c57bl/6 mice received vehicle (VEH) or fluoxetine (FLX; 20 mg/kg) for 15 consecutive days. Twenty-one days after the last exposure to FLX, mice were screened for side-preference bias (preconditioning), and were randomly assigned to receive cocaine (0, 2.5, 5, 10, or 20 mg/kg) for four consecutive days (conditioning). During the conditioning trials, mice were initially administered with saline and confined to the preferred compartment for 25 min. Three h later, mice were administered with cocaine, and confined to the opposite side of the chamber (25 min). On test day (preference), mice were given access to the entire apparatus (25 min), and the time spent in each compartment of the testing chamber was recorded.

Male c57bl/6 mice were randomly assigned to receive FLX (0 or 20 mg/kg) for 15 consecutive days, either during adolescence (PD 35–49) or adulthood (PD 65–79; see Fig. 2 for experimental timeline). FLX hydrochloride (Sigma-Aldrich, St. Louis, MO) was diluted in sterile double distilled water (vehicle; VEH), and administered in a volume of 2 ml/kg by intraperitoneal (IP) injection. The FLX dose and regimen was selected because it yields significant effects on behavior and gene expression^5^,^39^,^40^,^41^. Twenty-one days after FLX treatment (i.e., PD 70 for adolescent-pretreated and PD 100 for adult-pretreated mice), sensitivity to the rewarding properties of cocaine (0, 2.5, 5, 10, or 20 mg/kg) was assessed using the CPP paradigm (see below). Cocaine hydrochloride (Sigma-Aldrich) was diluted with sterile saline and administered in a volume of 2 ml/kg by IP injection.

### Conditioned place preference (CPP) procedure

Place preference conditioning was carried out as previously described^42^, using a three-compartment apparatus^12^, where compartments differed in floor texture and wall coloring. On the preconditioning day, mice were allowed to freely explore the entire apparatus for 25 min to obtain baseline preference to any of the three compartments (side compartments: 23 × 16 × 36 cm; middle compartment: 9 × 16 × 36 cm, L × W × H). Conditioning trials (25 min, two per day) were given on four consecutive days. During the conditioning trials, mice received an IP saline injection (2 ml/kg) and were confined to the preferred compartment of the apparatus (biased procedure^15^). After 3 h, mice received cocaine (0, 2.5, 5, 10, or 20 mg/kg, IP) and were confined to the opposite (non-preferred) side compartment. On test day (preference), mice were again allowed to freely explore the entire apparatus for 25 min (i.e., PD 75 for mice that received FLX-pretreatment during adolescence, and PD 105 for mice that received FLX-pretreatment as adults).

### Data analysis

Separate two-way ANOVAs, with FLX (pretreatment) and cocaine (post-treatment) as sources of variance, were conducted for adolescent and adult FLX-pretreated groups. This approach was taken in order to avoid age-specific influences on locomotor activity between the groups. Tukey post hoc tests were used to examine all pairwise comparisons. Planned comparisons were also conducted to examine the hypothesis that FLX pretreatment will enhance cocaine-induced reward. Statistical significance was defined as *p* < 0.05. Data are presented as mean + s.e.m.

## Additional Information

**How to cite this article**: Iñiguez, S. D. *et al.* Fluoxetine exposure during adolescence increases preference for cocaine in adulthood. *Sci. Rep.* **5**, 15009; doi: 10.1038/srep15009 (2015).
